# Eye Fixation-Related Potentials during Visual Search on Acquaintance and Newly-Learned Faces

**DOI:** 10.3390/brainsci11020218

**Published:** 2021-02-10

**Authors:** Seungji Lee, Doyoung Lee, Hyunjae Gil, Ian Oakley, Yang Seok Cho, Sung-Phil Kim

**Affiliations:** 1 Brain-Computer Interface Laboratory, Department of Biomedical Engineering, Ulsan National Institute of Science and Technology, Ulsan 44919, Korea; seungjilee@unist.ac.kr; 2 Interaction Laboratory, Department of Design, Ulsan National Institute of Science and Technology, Ulsan 44919, Korea; dylee@unist.ac.kr (D.L.); rlfguswo@unist.ac.kr (H.G.); ianoakley@unist.ac.kr (I.O.); 3 Human Performance Laboratory, Department of Psychology, Korea University, Seoul 02841, Korea; yscho_psych@korea.ac.kr

**Keywords:** visual search, familiarity, eye-fixation related potential, eye-tracking, face

## Abstract

Searching familiar faces in the crowd may involve stimulus-driven attention by emotional significance, together with goal-directed attention due to task-relevant needs. The present study investigated the effect of familiarity on attentional processes by exploring eye fixation-related potentials (EFRPs) and eye gazes when humans searched for, among other distracting faces, either an acquaintance’s face or a newly-learned face. Task performance and gaze behavior were indistinguishable for identifying either faces. However, from the EFRP analysis, after a P300 component for successful search of target faces, we found greater deflections of right parietal late positive potentials in response to newly-learned faces than acquaintance’s faces, indicating more involvement of goal-directed attention in processing newly-learned faces. In addition, we found greater occipital negativity elicited by acquaintance’s faces, reflecting emotional responses to significant stimuli. These results may suggest that finding a familiar face in the crowd would involve lower goal-directed attention and elicit more emotional responses.

## 1. Introduction

Suppose that we want to find someone in the crowd whom we have never met before, but know their faces by photos given in advance. Although we will be able to identify the target person easily by utilizing the newly acquired knowledge, it would be different from the case when we find the face of our acquaintance in the crowd. We would most likely put less effort to find a familiar face than an unfamiliar face. However, it remains elusive how we devote attention in finding such a familiar face. Two types of attentional processes may interplay during visual search to maintain the efficiency of our visual processing [1,2,3,4]. One is exogenous, stimulus-driven attention directed by the characteristics of visual stimuli, and the other is endogenous, goal-directed attention driven by one’s intention to find a target. Hence, it is of our interest to understand the roles of different attentional processes in searching for a familiar face among others.

Many event-related potential (ERP) studies have investigated neural activity during face recognition in order to understand attentional processes related to the rapid processing of facial information [5,6,7,8,9]. The ERP results have suggested that our attention is attracted more by familiar faces compared to unfamiliar ones due to emotional significance. For example, the faces of relatives, friends, and romantic partners induced greater positivity of ERPs over frontal to occipital midline areas than celebrities or strangers did [6,7,8]. Another study found a difference in ERPs between acquaintances’ faces and newly-learned faces [5]. This study employed the oddball paradigm with strangers’ faces as frequent and acquaintance or newly-learned faces as infrequent stimuli. It showed a slower P300 latency in response to newly-learned faces than that in response to faces of acquaintances. It implies that the recognition of newly-learned faces is more cognitively demanding than that of familiar faces. Yet, this study, as well as previous ERP studies, adopted a stimulus-response paradigm with passive viewing where subjects were shown a series of face stimuli so that their attention was largely driven by the intrinsic properties of faces. Therefore, it is still not clear how familiarity modulates goal-directed attention towards faces. To address this question, other types of paradigms, such as a visual search task, may help us find the effect of familiarity on goal-directed attention towards a target face.

In a visual search task, not only are behavioral performance measures such as accuracy and reaction time [3,10,11] used widely, but also eye gaze measures have been used increasingly to examine visual attention [12,13,14,15]. For instance, Calvo and Nummenmaa [12] revealed that subjects found a happy face with a faster fixation onset than other emotional faces, indicating a stronger stimulus-driven attentional orientation to happy faces. In addition, Riby and Hancock [14] suggested a lack of attention towards socially relevant information (i.e., face) in patients with autism, indicated by reduced gazes at faces.

To associate neural activity with eye gaze behavior during visual search, researchers have simultaneously measured electroencephalography (EEG) and eye movements, extracting brain activity that is time-locked to eye fixation, called eye fixation-related potentials (EFRPs) [16,17,18,19,20]. Recent studies on EFRPs revealed that prominent components found in ERPs also appeared in EFRPs during visual search tasks. For instance, visual search for a simple stimulus (i.e., shape or color) among similar distractors [17] or for a target item (e.g., a cup hanger) in realistic scenes [16] elicited P300 in EFRPs. Moreover, Kaunitz and Kamienkowski [18] has demonstrated that visual search for a human face in the oddball paradigm induced P300 in EFRPs as well.

By measuring and analyzing EFRPs in a visual search task, this study aimed to investigate how familiarity affects behavioral and neural responses related to stimulus-driven attention and goal-directed attention towards a face. The familiarity with a face was manipulated by the time of acquaintance by using the visual images of acquaintances’ faces (i.e., more familiar) and newly-learned faces (i.e., less familiar). Since little was known about EFRPs in relation to a visual search for a face, we assumed that EFRP waveforms would be similar to ERP waveforms related to face recognition in the oddball paradigm [16,18]. Accordingly, we expected that the P300 component would be elicited in EFRPs by the detection of a target face regardless of whether the face is of an acquaintance or newly learned.

In this study, participants searched for either an acquaintance’s face or a newly- learned face as a target among other distracting faces. As such, participants’ attention towards a target face was driven by the task goal, as well as the characteristics of facial stimuli. Thus, we expected two EFRPs related to stimulus-directed attention and goal-directed attention. One was the late positive potential (LPP) over frontal to parietal midline, reflecting stimulus-directed attention driven by emotionally significant faces [5,6,7,8,9]. We expected that acquaintances’ faces would elicit larger LPPs than newly-faces would. The other was enhanced positivity appearing 500–700 ms after stimulus onset over the parietal area reflecting goal-directed attention [19,20]. We expected that this positivity would be increased by newly-learned faces as more efforts are needed for searching for newly-learned faces than acquaintances’ faces.

## 2. Materials and Methods

### 2.1. Participants

Eighteen healthy university students with normal or corrected-to-normal vision with reportedly no neurological disorders participated in the study. Each of them was recruited to belong to one of the two groups. The acquaintance (AQ) group consisted of nine participants (6 female, mean age of 23.89 ± 1.97 years old) who personally knew the target faces for a long time (>1 years), whereas the newly-learned (NL) group consisted of the other nine participants (4 female, mean age of 20.56 ± 2.41 years old) who had not known the target faces prior to the experiment. 

### 2.2. Stimuli

Two different sets of facial images were created. The first set included the facial images of 28 persons whom the AQ group knew before (SET1), and the second set included the facial images of 28 persons whom neither the AQ nor NL group knew before (SET2). There were 14 female faces in each of SET1 and SET2, respectively. Participants in the AQ group was instructed to choose 4 target faces in SET1. Participants in the NL group were also instructed to choose 4 target faces in SET1, although they had not known those faces before the experiment. Then, participants in both groups were trained to search the target faces in a preliminary session without EEG recordings to become familiar with the visual search task as well as the target faces. Thus, there were four conditions in this study: Target and AQ, target and NL, non-target and AQ, and non-target and NL. We aimed to find whether two groups (AQ and NL) exhibited different neural responses on a familiar face in the visual searching task by comparing the ‘target and NL’ and ‘target and AQ’ conditions and to confirm that the two groups did not have a different strategy for finding faces (i.e., no significant difference in behavioral and neural responses between the ‘non-target and NL’ and ‘non-target and AQ’ conditions).

We verbally asked participants in the NL group whether they knew the faces in SET1 (i.e., target) before the main experiment, and confirmed that no one in the NL group knew those faces in SET1 beforehand. Those shown in SET1 and the AQ group belonged to the same department in the same university. Each subject in the AQ group chose 4 among 28 faces whom they had known for more than a year. As people whose facial images were used in SET2 (i.e., non-target) were the students in the other university, we assumed that participants would not know their faces. To verify this, we asked all participants in each AQ and NL group to report any acquaintance in SET2 after the main experiment and received no such a report at all.

**Figure 1:**
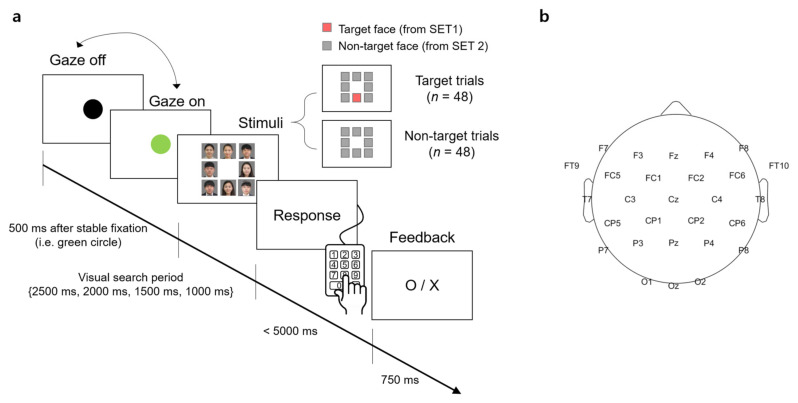
An experimental task and an electroencephalography (EEG) montage. (**a**) Timeline of stimulus presentation. The face images were blurred for privacy reason. (**b**) The EEG montage used in this study.

A visual stimulus presented to participants contained 8 facial images that were arranged in the form of a 3 × 3 matrix with the center slot empty (Figure 1a). A facial image was the color photo of a person from the chest up. The size of the image was 350 × 350 pixels surrounded by 5 pixels of white padding, thereby there was a 10-pixel interval between the images. The resolution of the screen was 1920 × 1080 (W × H) pixels.

For each trial of the visual search task, the stimulus contained either a target face from SET1 and 7 unknown faces from SET2 (denoted as a “target trial” hereafter) or 8 unknown faces from SET2 only (denoted as a “non-target trial” hereafter). The target face and/or unknown faces were randomly selected from the sets in each trial. The spatial arrangement of the facial images at each trial was also randomized.

### 2.3. Task

Participants sat approximately 1.5 m in front of a screen (145 × 81 cm^2^) and were instructed to minimize body and head movements during the experiment. The visual stimulus was displayed on the screen by the projector (Profix 1000, VPixx technologies, Canada, frame rate = 120 Hz). At the beginning of a trial, participants were required to fixate their gaze at a black circle located at the center of the white screen. At any moment the fixation was detected on the circle by the eye tracker, the black circle turned green. If the circle remained green over 500 ms, it disappeared and a visual search period started in which the visual stimulus of the facial image matrix was shown to initiate the search task. In the visual search period, participants searched for a target face. After the search period ended, the word, “Response” appeared on the screen to inform participants to indicate the location of a target face in the matrix by pressing one of the 9 keys on the numerical keypad (i.e., from “1” to “9” in the numerical keypad on the computer keyboard). Using this numerical keypad could provide a direct spatial mapping between the locations of each facial image and their corresponding correct key. If participants determined that they could not find the target, they pressed the center key (i.e., ‘5′) on the keypad. No response was recorded if participants failed to press any key within 5000 ms after the response period began. A trial ended by showing visual feedback for correctness given as “O” or “X” on the screen for 750 ms immediately after the key-pressing response. Then, the black circle appeared again to begin with the next trial. An inter-trial interval varied across participants due to an individual difference to stabilize gaze at the fixation point (i.e., black circle). The duration of the visual search period was set to 2500, 2000, 1500, or 1000 ms. The trials with the same duration were grouped into a single block and there were four separate blocks according to four different durations. The total number of trials per block was 24, with 12 target trials and 12 non-target trials. The presentation order of the target and non-target trials was randomized within a block. The order of the four blocks was also randomized across participants.

### 2.4. Behavioral Data Analysis

A correct rate was calculated in each block as a ratio of the number of trials with correct answers to the total number of trials. In addition, errors in the target trials were categorized into two types according to a failure to detect target presence (Error 1) or a wrong location of the target face (Error 2). We assessed the effect of the visual search period duration on the total correct rate (i.e., the correct rate on both target and non-target trials) using a one-way repeated measures ANOVA (rmANOVA). If rmANOVA revealed blocks that exhibited significantly inferior performance compared to others (*p* < 0.05), we excluded them from the analysis. Afterward, all the data collected in the remaining blocks were combined together for the subsequent analyses.

### 2.5. Eye-Tracking Data Acquisition and Analysis

During the entire experiment, the eye movements of participants were recorded using an eye-tracker system (EyeLink 1000, SR Research, Ottawa, ON, Canada) at a sampling rate of 500 Hz. The 2D coordinates of the left and right eyes on the screen were obtained and averaged in real-time using MATLAB 2017a (Mathworks, Natick, MA, USA). The average eye-coordinate data were then fed to a custom-made Arduino program that was developed in-house to run the experiment.

The recorded eye gaze data were analyzed based on areas of interest (AOIs). Eight AOIs were defined as the eight cells in the 3 × 3 facial image matrix described above, excluding the center cell. The fixation on an AOI was measured using a velocity-based detection algorithm with λ = 15, a parameter for tuning a saccade detection threshold [21]. The setup of λ = 15 provided robust and stable saccade detection relative to the noise level in the eye velocity signals of a single trial. Any points between two saccades were considered as a fixation. Then, a fixation event specific to our analysis was defined as either the first fixation on the AOI containing the target face for the target trial or the first fixation on the AOI with the longest fixation for the non-target trial. Therefore, the fixation event occurred once for every non-target trial, or once for those target trials where the target face was correctly identified.

For each fixation event, we defined fixation onset time as the time elapsed from stimulus onset to the onset of the fixation event, fixation duration as the time elapsed from the first entry to an AOI to the first exit from that AOI, and dwell time as the sum of total fixation time on an AOI.

### 2.6. EEG Data Acquisition and Analysis

EEG signals were acquired using 31-channel wet Ag/Cl electrodes (actiCHamp, Brain products GmbH, Germany) at a sampling rate of 500 Hz. The acquired EEG signals were band-pass filtered with 0.05-Hz and 100-Hz cutoff frequencies using a finite impulse response (FIR) filter. The position of 31 electrodes was determined following the 10/20 international system: FP1, FPz, FP2, F7, F3, Fz, F4, F8, FT9, FC5, FC1, FC2, FC6, FT10, T7, C3, Cz, C4, T8, CP5, CP1, CP2, CP6, P7, P3, Pz, P4, P8, O1, Oz, and O2. An additional electrode was attached to the left mastoid as a ground and the right one as a reference (Figure 1b).

EEG preprocessing was performed as follows. First, to remove line noise and high-frequency noise, the EEG signals were bandpass filtered again within a frequency band of 0.1–55 Hz using an FIR filter. Second, independent component analysis (ICA) was applied to the EEG signals to remove eye movement artifacts. Third, the EEG signals were re-referenced by the common average reference (CAR) method. Finally, the EEG signals were low-pass filtered with a high-frequency cutoff of 30 Hz to reduce the effect of high-frequency components of EEG on EFRPs.

An epoch of the EEG analysis was extracted from each trial 200 ms before and 800 ms after the fixation onset time. The baseline period of the EFRP was set to be −200~0 ms, where 0 ms denotes the fixation onset time. The EEG signal in each epoch was corrected to the baseline by subtracting the mean of EEG amplitudes within the baseline from the entire EEG amplitudes in the epoch. Any trial with a peak deflection exceeding ±80 μV was excluded from averaging. The EFRPs for each condition of target presence (i.e., target vs. non-target trials) or familiarity (AQ vs. NL) were obtained by averaging the EEG signals over epochs with correct responses. It resulted in four EFRPs for each channel and participant, corresponding to four conditions: Target and AQ, target and NL, non-target and AQ, and non-target and NL. Additionally, those participants who did not exhibit a common EFRP component, namely P100 at the visual cortex (i.e., O1, Oz, O2), were excluded from the subsequent analysis [22]. This last procedure excluded one participant from each group out of our analysis.

### 2.7. Statistical Analysis

We examined the effects of the presence of a target face and the type of participant groups on behavioral, eye gaze, and EEG data, respectively. In our experimental design, the presence of a target face constituted a within factor, whereas the participant group (i.e., familiarity) was a between factor. The target presence factor consisted of two levels, presence and absence of the target face, and the familiarity factor consisted of two levels, the AQ and the NL group.

For the behavioral data analysis, two-way mixed repeated measures ANOVA (rmANOVA) was conducted on the correct rate with target presence and familiarity as two factors. In addition, a two sample t-test was used to evaluate differences in Error 1 and Error 2 between the groups, respectively. Similarly, for the eye gaze data analysis, two-way mixed rmANOVA was conducted on the fixation onset time, fixation duration, and dwell time, respectively.

For the EEG data analysis, we first reduced the number of channels from 31 to 28, excluding prefrontal sites: Fp1, Fpz, and Fp2 due to high noise from eye movements. Again, two-way mixed rmANOVA was conducted on the EFRP amplitude time-averaged within a 50-ms non-overlapping window sliding from 0 to 800 ms, similar to the procedure of the previous EFRP study [23]. After each rmANOVA, a multiple comparison test with Bonferroni correction was conducted to adjust the *p*-value: Denoted hereafter as $p_{b}$.

## 3. Results

### 3.1. Behavior Results

As to the influence of the search duration on behavioral performance, a one-way repeated measures ANOVA (rmANOVA) revealed the significant main effect of the visual search period ($F_{3,51}$ = 8.22, *p* = 0.0001) on the correct rate. Multiple comparison with Bonferroni correction indicated that the correct rate of target detection in the 1 s period was significantly lower than those of 2 s ($p_{b}$ < 0.001) and 1.5 s ($p_{b}$ = 0.009) periods, respectively, but not lower than 2.5 s ($p_{b}$ > 0.2). Furthermore, we examined whether the correct rate in the target trials was different between the NL and AQ groups in the 1 s period because it could be more challenging to find a target face with less familiarity during a brisk stimulus presentation. The AQ group, however, showed a lower correct rate on 1 s period block (0.68 ± 0.13) than the NL group (0.82 ± 0.12) did ($t_{16}$ = −2.52, *p* = 0.023). This was derived from increased Error 1 in the AQ group (the AQ group missed a target more frequently than the NL group, 0.30 ± 0.13 > 0.16 ± 0.09, $t_{16}$ = 2.62, *p* = 0.018). On the other hand, there was no significant difference in Error 2 between the groups (*p* = 0.69). Accordingly, we excluded the 1 s period block from subsequent behavior, eye-tracking, and EEG analyses.

**Figure 2:**
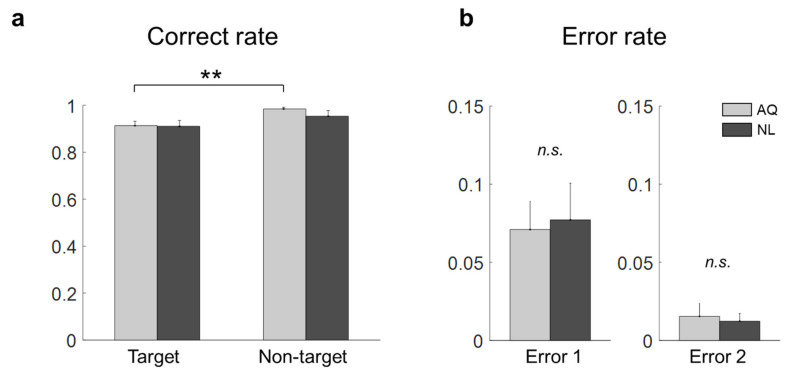
Behavior results of the target face detection task. (**a**) Correct rates for all the conditions (a double star denotes $p_{b}$ < 0.01). (**b**) Error 1 and Error 2 rates of the AQ (Acquaintances faces) and NL (Newly-learned) groups (*n*.*s*. denotes not significant).

Without the 1 s period block, we compared the correct rates of target and non-target trials of the AQ and NL groups. Two-way mixed rmANOVA revealed the significant main effect of target presence ($F_{1,16}$ = 12.36, *p* = 0.002, $\eta_{p}{}^{2}=0.44$), but not of familiarity ($F_{1,16}$ = 0.56, *p* = 0.46), on the correct rate. No interaction effect was found ($F_{1,16}$ = 0.73, *p* = 0.41). As shown in Figure 2a, the correct rate for the non-target trials (0.98 ± 0.01) was significantly higher than that for the target trials (0.91 ± 0.02) in the AQ group ($p_{b}$ = 0.007). No difference was found between the AQ and NL groups with respect to both Error 1 ($t_{16}$ = −0.21, *p* = 0.84) and Error 2 ($t_{16}$ = 0.32, *p* = 0.24).

### 3.2. Eye-Tracking Results

**Figure 3:**
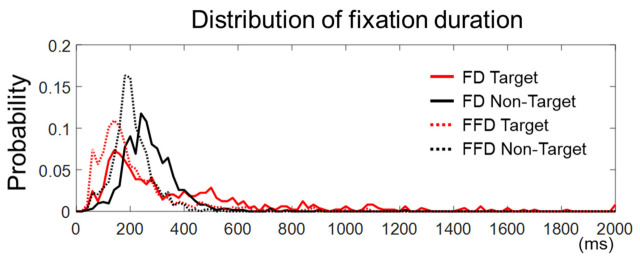
The distribution of fixation duration. The solid lines represent fixation durations (FD) defined as the time elapsed from the first entry to an AOI to the first exit from that AOI. The dashed lines stand for first fixation durations (FFD) defined as the duration of a first fixation point between saccades in an AOI. The onset of fixation in target trials is the first fixation on an AOI and that in non-target trials is the longest fixation on non-target faces.

The fixation duration was defined in this study as the time elapsed from the first entry on an AOI to the first exit out of that AOI. Yet, there could be more than one fixation in an AOI. Hence, we examined fixations within the target face in the target trials or within the longest-dwelled non-target face in the non-target trials to see if there were more than one fixation in the AOIs. We found that the number of target trials with more than one fixation was 3.87 ± 2.45 out of 36, and the number of non-target trials with more than one fixation was 6.25 ± 4.37 out of 36. Many of these trials were not rejected by peak amplitudes (see Section 2.6). Note that the number of accepted target trials with a single fixation was 25.19 ± 3.99 and that of non-target trials was 27.00 ± 3.94. We also compared the distribution of all the fixation durations and that of the first fixation durations (in case more than one fixation existed) for both the target and non-target trials, respectively (Figure 3). We observed that the cumulative distribution function of the first fixation durations was larger than that of all fixation durations, for both the target and non-target trials, indicating that the first fixation durations were statistically shorter (two samples Kolmogorov–Smirnov test, k = 0.30 for target and k = 0.37 for non-target trials, *p* < 0.0001). This implies that the later components of EFRPs on the first fixation might include the early components of EFRPs based on the subsequent fixation. There were, however, only a few accepted epochs with multiple fixations for the EFRP analysis (3.87 ± 2.45 for the target trials and 6.25 ± 4.37 for the non-target trials). In brief, although the first fixations exhibited relatively shorter durations than pre-defined fixations in Section 2.5, calculated EFRPs included only a few trials with such multiple fixations, in addition to much more trials with a single fixation on the AOIs.

**Figure 4:**
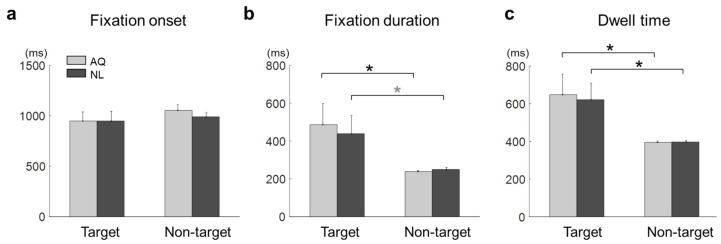
Eye-tracking results of the target face detection task. (**a**) Fixation onset, (**b**) fixation duration, and (**c**) dwell time on the target faces (Target) or non-target faces with the longest fixation (Non-garget) for the AQ (Acquaintances faces) and NL (Newly-learned) groups. The stars indicate the significance from a multiple comparison test: Black stars denote $p_{b}$ < 0.05 and a gray one denotes $p_{b}$ < 0.1.

The mean fixation onset time on the target faces was 948.41 ± 272.50 ms and that on the non-target faces with the longest fixation was 1021.87 ± 149.26 ms. The mean fixation onset time in the AQ group was 1001.15 ± 225.00 ms and that in the NL group was 969.13 ± 219.60 ms. A two-way mixed rmANOVA on fixation onset time revealed neither main effects of target presence (F_1,16_ =1.03, *p* = 0.32) and familiarity (F_1,16_ = 0.17, *p* = 0.68) nor interaction effect (F_1,16_ = 0.20, *p* = 0.66) (Figure 4a).

The mean fixation duration was 462.17 ± 304.96 ms on the target faces and 244.29 ± 23.96 ms on the non-target faces with the longest fixation. The mean fixation duration of the AQ group was 362.35 ± 263.96 ms and that of the NL group was 344.11 ± 211.03 ms. A two-way mixed rmANOVA on fixation duration revealed the main effect of target presence (F_1,16_ = 9.22, *p* = 0.007, $\eta_{p}{}^{2}$ = 0.37), but not of familiarity (F_1,16_ = 0.06, *p* = 0.81). No interaction effect was found (F_1,16_ = 0.16, *p* = 0.69). Multiple comparison with Bonferroni correction indicated that the AQ group gazed at the target faces significantly longer than the non-target faces ($p_{b}$ = 0.027), whereas the NL group gazed at the target faces marginally longer than the non-target faces ($p_{b}$ = 0.081) (Figure 4b).

The mean dwell time on the target faces was 634.78 ± 287.09 ms and that on non-target faces with the longest fixation was 395.98 ± 21.25 ms. The mean dwell time of the AQ group was 512.61 ± 259.37 ms and that of the NL group was 509.15 ± 213.79 ms. A two-way mixed rmANOVA on dwell time showed the significant main effect of target presence (F_1,16_ = 12.54, *p* = 0.003, $\eta_{p}{}^{2}$ = 0.44), but not of familiarity. The target faces evoked longer dwell time than the non-target faces for both the AQ ($p_{b}$ = 0.017) and NL groups ($p_{b}$ = 0.032) (Figure 4c).

### 3.3. EEG Results

**Figure 5:**
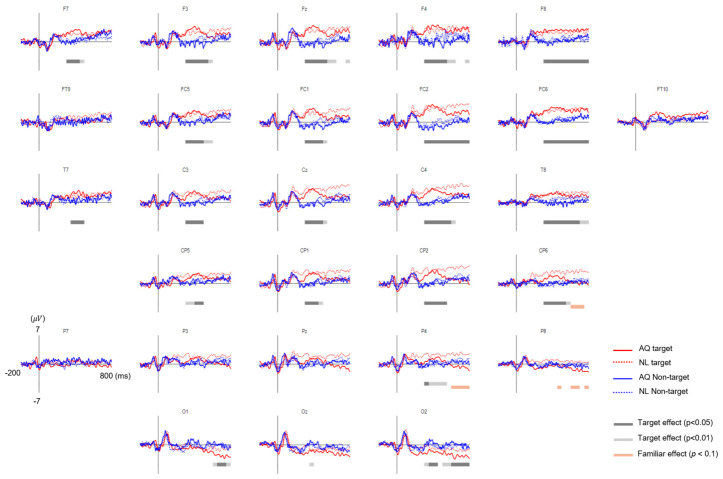
The grand average eye fixation-related potentials (EFRPs) of searching faces by AQ (Acquaintances faces, solid line) and NL group (Newly-learned faces, dash line). Red line represents when they looked at target faces and blue when looking at non-target faces. The bars below EFRPS indicates significant main effect of target presence (dark gray for *p* < 0.05 and light gray for *p* < 0.01) and group (orange for *p* < 0.01) resulting from two-way mixed repeated measures ANOVA (rmANOVA) for averaged amplitude for every 20 ms time window.

The grand average EFRPs elicited by the fixation events in the AQ and NL groups are presented in Figure 5. The amplitudes of the earlier EFRP component (i.e., P1) were not different across all the conditions.

**Figure 6:**
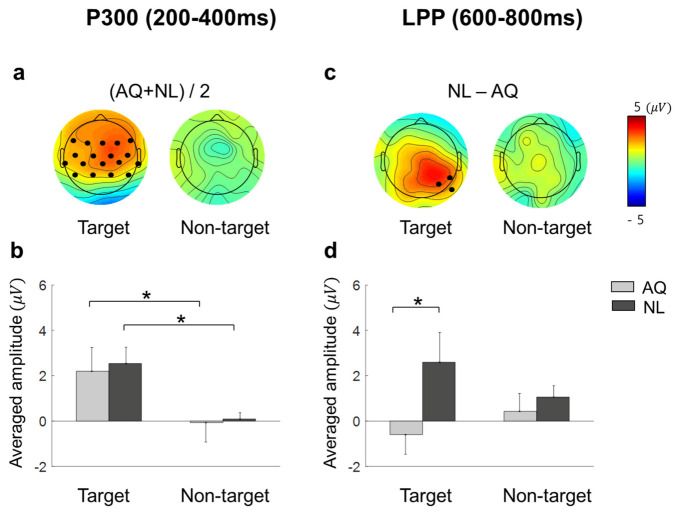
The scalp distributions and the mean amplitude of EFRPs: P300 and late positive potential (LPP). (**a**) Scalp distributions for the target and non-target trials of the average of AQ (Acquaintances faces) and NL (Newly-learned). Black dots indicate the channels where a significant main effect of target presence was found resulting from two-way mixed rmANOVA on P300. (**b**) Mean amplitude of the channels indicated in (**a**). (**c**) Scalp distributions for the difference between AQ and NL group of target and non-target trials. Black dots indicate the channels where a significant main effect of group resulting from two-way mixed rmANOVA on LPP. (**d**) Mean amplitude of the channels indicated in (**c**). A star in the bar plots represents $p_{b}$ < 0.05.

Approximately 200 ms after fixation onset, the target faces elicited significantly enhanced positivity for both AQ and NL groups over widely distributed channels, including: F7, F3, Fz, F4, F8, FC5, FC1, FC2, FC6, T7, C3, Cz, C4, T8, CP5, CP1, CP2, and CP6 (Figure 6a). This positive deflection was considered as the P300 component. Then, we obtained the mean P300 amplitude by calculating the time-average of amplitude within a window of 200–400 ms post-stimulus for each channel. These mean P300 amplitude values were averaged again across the 17 channels selected above. Lastly, we statistically analyzed the effect of target presence and familiarity on this averaged P300 amplitude. Two-way mixed rmANOVA revealed the main effect of target presence ($F_{1,14}$ = 10.72, *p* = 0.005, $\eta_{p}{}^{2}$ = 0.43) on the P300 amplitude. Multiple comparison with Bonferroni correction showed a larger P300 amplitude for the target faces than for the non-target faces in both AQ and NL groups ($p_{b}$ = 0.043 and $p_{b}$ = 0.031, respectively) (Figure 6b). Neither main effect of familiarity (*p* = 0.77) nor interaction effect (*p* = 0.90) was found.

Approximately 600 ms after fixation onset, which was considered as the latency of late positive potential (LPP), a marginal amplitude difference in the positive EFRP amplitudes between the groups was found at CP6, P4, and P8. Similar to P300, we obtained the time-averaged LPP amplitudes within a window of 600–800 ms per channel and averaged them across 3 channels selected above. Two-way mixed rmANOVA showed the main effect of familiarity on the LPP amplitude ($F_{1,14}$ = 3.65, *p* = 0.077, $\eta_{p}{}^{2}$ = 0.21) (Figure 6d). The LPP amplitude of the NL group was larger than that of the AQ group in the target trials ($p_{b}$ = 0.016), but not in the non-target trials ($p_{b}$ = 0.66), resulting from multiple comparison with Bonferroni correction. The main effects of target presence and interaction effect were not found (*p* = 0.76 and *p* = 0.14, respectively).

**Figure 7:**
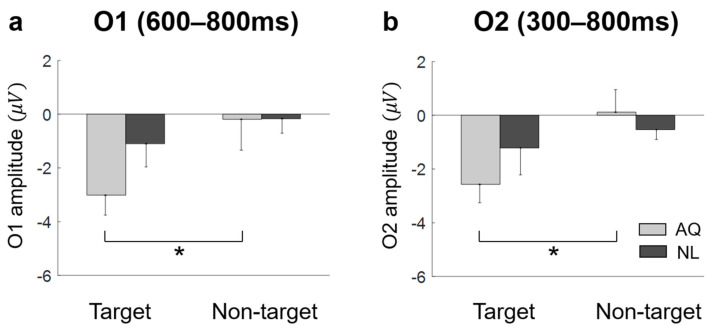
The amplitude at bilateral occipital area. (**a**) AQ (Acquaintances faces) group had significantly negative value in target trials then non-target trials at O1 during 600–800 ms. (**b**) at O2 during 300–800 ms. A star represents $p_{b}$ < 0.05.

Lateral occipital areas (i.e., O1, O2) showed different patterns of EFRPs from those in other channels (see Figure 5). AQ group elicited more negative deflection in these occipital areas than the NL group did. For O1, two-way mixed rmANOVA showed the significant main effect of target presence on the mean EFRP amplitude in the window of 600–800 ms (F_1,14_ = 5.32, *p* = 0.037, $\eta_{p}{}^{2}$ = 0.28). For O2, two-way mixed rmANOVA also showed the significant main effect of target presence on the mean EFRP amplitude in the window of 300–800 ms (F_1,14_ = 5.91, *p* = 0.029, $\eta_{p}{}^{2}$ = 0.30). The multiple comparison analysis with Bonferroni correction showed significantly lower EFRP amplitudes for the target faces than for the non-target faces at both channels (O1: *p* = 0.028 and O2: *p* = 0.016) in the AQ group, which was not observed in the NL group (Figure 7).

## 4. Discussion

The present study aimed to find the effect of familiarity with a person on behavioral and neural activities in searching for their face among distracting faces. Specifically, participants either personally knew the target faces (AQ group) or newly learned the faces before an experiment to become familiar with visual images of the target faces (NL group). Hence, any difference in behavioral and neural activities between the groups would be related to personal relationships with target persons, not mere familiarity with visual images. The behavioral analyses revealed that familiarity with persons did not have advantages in behavioral performance to search for their faces in the crowd compared to the case when participants newly learned the faces and searched for them. Both the AQ and NL groups exhibited similar searching accuracy and gaze behavior. In contrast, familiarity induced differences in EFRPs over the right parietal and occipital areas. We found two EFRP components modulated by familiarity when searching for a target face: (1) Larger right parietal LPP amplitude in the NL group compared to the AQ group; and (2) enhanced negativity at the occipital area in the AQ group compared to the NL group. Our results suggest that although one could learn to search for someone they did not know by face as quickly and accurately as another who previously knew the target person, their neural responses were dissimilar due to attentional and emotional differences.

Some behavioral performances, such as correct rates, were not different between the AQ and NL groups. We speculate that the training session before the main search task improved the searching performance of those who newly learned faces (NL group) to the level of those who did not need to learn faces, but were personally familiar with the faces (AQ group). On the other hand, target presence made a difference in behavior; searching accuracy was lower in the target trials than in the non-target trials. It might be related to the fact that there could be two different types of error (false targeting and missing a target) in the target trials, while only one possible error (a failure to reject target presence) could be made in the non-target trials. In the 1 s period block, however, the AQ group missed a target more frequently than the NL group, yet they were supposed to perform better. This may imply that those who were personally familiar with the faces were less involved in the searching task, as they were more familiar with the target faces. The result of longer fixation duration and dwell time on target faces than non-target faces shown in our study is consistent with many previous studies [16,20,24,25].

Neural correlates of target detection have been explored in many EFRP studies [16,17,18,20,25,26,27]. Especially, Kaunitz and Kamienkowski [18] showed that a P300-like component was elicited from EFRPs by the detection of a target face in both the visual search and the oddball tasks. Consistent with these previous studies, we observed larger P300 amplitudes when participants fixated on a target face than when they fixated on a non-target face. This may indicate that we can infer whether one is gazing at a target person or not from the presence of P300 in EFRPs.

The larger right parietal LPP in the NL group than in the AQ group may imply a difference in attentional processing after the detection of a target face. The right parietal cortex has been involved in voluntary attentional processes for target detection [27,28,29]. In particular, the right superior temporal sulcus was more activated when subjects should pay attention to identify a person by the face than when they perceived the direction of eye gaze [30,31]. There could be two other possibilities driving a difference in LPPs between the AQ and NL groups: The retrieval of personal memory and the emotional recognition of familiar faces [5,32,33]. Unlike our results, however, memory retrieval typically elicits LPPs in left parietal lobe (see Vilberg and Rugg [34] for review) and newly-learned faces would induce less parietal activity due to the lack of personal knowledge [32,33]. In addition, familiar faces induced larger frontal P3a that reflected emotional recognition [33]. However, our results showed LPPs in right parietal area, which was larger in response to newly-learned faces, but no difference in frontal P3a. Therefore, we suspect that the difference in right parietal LPPs between the groups might not be directly linked to memory retrieval and emotional recognition. Rather, larger right parietal LPPs after fixation on a newly-learned face than on an acquaintance’s face might reflect that participants implicitly paid more attention to a newly-learned face than an acquaintance’s face. Moreover, this putative increase in attention was more likely related to goal-directed attention than stimulus-driven attention as the latency and amplitude of P300 as well as behavioral responses including eye gaze to target faces were not distinguishable between the AQ and NL groups.

We speculate that participants needed to pay more goal-directed attention to a newly-learned face because it was cognitively more demanding to find a previously unknown, but newly-learned face than to find an acquaintance’s face. In line with this speculation, the NL group searched target faces better than the AQ group in a particularly difficult situation (i.e., 1 s period), implying that people who newly learned the faces were more engaged in the task than those who were familiar with the faces. Therefore, it involved more attention driven by the task goal of finding a target face. For the relatively simple visual search task in our study (finding one out of eight faces), one could achieve task performance by learning at a similar level to those who already knew the target person, but with spending more attentional resources.

On the other hand, we observed unexpected negative EFRPs at the occipital area in response to familiar faces. A number of studies demonstrated that task-relevant targets (e.g., words or items) induced larger positive EFRPs at the occipital area than task-irrelevant targets did [16,18,35]. In contrast, other studies showed that a target stimulus elicited more negative deflections than distractors did at the occipital area in a simple visual search task [17]. In our case, we observed a significant difference in negative occipital EFRPs between target and non-target stimuli only in response to acquaintances’ faces, which might indicate that larger occipital negativity was induced not simply by target detection. In fact, an ERP study reported larger negative deflections at the occipital area in response to emotional pictures [36]. Thus, our finding of negative EFRPs at the occipital area might reflect emotional responses to familiar faces.

Yet, the current study still has several limitations. First, we failed to ask participants to continuously fixate their eyes on a target face immediately after detecting it during the experiment, which could cause a potential overlap of an EFRP in response to the target face with EFRPs associated with subsequent fixations. Second, the reaction time collected in the experiment could not be used to examine the effect of familiarity because participants responded about target detection only after the visual search period was over. Instead, we analyzed the fixation onset on target faces, which showed no difference by familiarity. Third, we did not measure behavioral responses regarding emotional recognition of acquaintances’ faces, which could have supported our conjecture that familiar faces induced more emotional responses. Similarly, we did not particularize the acquaintances as friends or colleagues because the purpose of this study was to distinguish the searching process of emotion-evoking faces (i.e., acquaintance) from that of effort-evoking faces (i.e., newly-learned) rather than more emotion-evoking faces from less emotion-evoking faces. In our future studies, we will pursue to address these issues to deepen our understanding of how our cognitive and neural processes operate in searching for someone we are familiar with.

Finally, our findings could contribute to expanding our knowledge of the searching process of familiar faces. In addition, our study may add value to the clinical research fields. For instance, future work can aim to find electrophysiological responses in patients with problems of face recognition [37] using EFRPs, as it has been shown that visual evoked potentials (VEPs) with reduced amplitude and delayed latency reflect the dysfunction in visual processing [38]. Moreover, it would be plausible to associate specific characteristics of EFRPs during visual processing with more biological mechanisms to make integrated accounts for neurodegenerative disorders such as retinal dystrophies [39,40].

## 5. Conclusions

In the present study, we investigated the effect of familiarity (acquaintances versus newly-learned) on attentional processes by exploring EFRPs and eye gazes when humans searched for faces among other distracting faces. Task performance and gaze behavior were indistinguishable for identifying either faces. Likewise, a P300 component representing a successful search of targe faces were equally detected for both acquaintances and newly-learned faces. On the other hand, we found that a personally familiar face in the crowd would involve lower goal-directed attention (i.e., small right parietal LPP) and elicit more emotional responses (i.e., large occipital activity). This study contributes to EFRP research field on visual search in that we could observe goal-directed and stimulus-driven cognitive process as well as typical brain activity during searching task.

## Data Availability

Data sharing is not applicable to this article.

## References

[1] Ferrari V., Codispoti M., Cardinale R., Bradley M.M. (2008). Directed and motivated attention during processing of natural scenes. J. Cogn. Neurosci..

[2] Müller H.J., Krummenacher J. (2006). Visual search and selective attention. Vis. Cogn..

[3] Wolfe J.M. (1994). Guided search 2.0 a revised model of visual search. Psychon. Bull. Rev..

[4] Yantis S. (1993). Stimulus-driven attentional capture and attentional control settings. J. Exp. Psychol..

[5] Bobes M.A., Quiñonez I., Perez J., Leon I., Valdés-Sosa M. (2007). Brain potentials reflect access to visual and emotional memories for faces. Biol. Psychol..

[6] Burdwood E.N., Simons R.F. (2016). Pay attention to me! Late ERPs reveal gender differences in attention allocated to romantic partners. Psychophysiology.

[7] Grasso D.J., Moser J.S., Dozier M., Simons R. (2009). ERP correlates of attention allocation in mothers processing faces of their children. Biol. Psychol..

[8] Grasso D.J., Simons R.F. (2011). Perceived parental support predicts enhanced late positive event-related brain potentials to parent faces. Biol. Psychol..

[9] Meijer E.H., Smulders F.T., Merckelbach H.L., Wolf A.G. (2007). The P300 is sensitive to concealed face recognition. Int. J. Psychophysiol..

[10] Schubö A., Gendolla G.H., Meinecke C., Abele A.E. (2006). Detecting emotional faces and features in a visual search paradigm: Are faces special?. Emotion.

[11] Tong F., Nakayama K. (1999). Robust representations for faces: Evidence from visual search. J. Exp. Psychol..

[12] Calvo M.G., Nummenmaa L. (2008). Detection of emotional faces: Salient physical features guide effective visual search. J. Exp. Psychol..

[13] Calvo M.G., Marrero H. (2009). Visual search of emotional faces: The role of affective content and featural distinctiveness. Cogn. Emot..

[14] Riby D.M., Hancock P.J. (2009). Do faces capture the attention of individuals with Williams syndrome or autism? Evidence from tracking eye movements. J. Autism Dev. Disord..

[15] Simpson E.A., Buchin Z., Werner K., Worrell R., Jakobsen K.V. (2014). Finding faces among faces: Human faces are located more quickly and accurately than other primate and mammal faces. Attention. Percept. Psychophys..

[16] Devillez H., Guyader N., Guérin-Dugué A. (2015). An eye fixation–related potentials analysis of the P300 potential for fixations onto a target object when exploring natural scenes. J. Vis..

[17] Kamienkowski J.E., Ison M.J., Quiroga R.Q., Sigman M. (2012). Fixation-related potentials in visual search: A combined EEG and eye tracking study. J. Vis..

[18] Kaunitz L.N., Kamienkowski J.E., Varatharajah A., Sigman M., Quiroga R.Q., Ison M.J. (2014). Looking for a face in the crowd: Fixation-related potentials in an eye-movement visual search task. NeuroImage.

[19] RÄMÄ P.I.A., Baccino T. (2010). Eye fixation–related potentials (EFRPs) during object identification. Vis. Neurosci..

[20] Wenzel M.A., Golenia J.-E., Blankertz B. (2016). Classification of Eye Fixation Related Potentials for Variable Stimulus Saliency. Front. Neurosci..

[21] Engbert R., Kliegl R. (2003). Microsaccades uncover the orientation of covert attention. Vis. Res..

[22] Kazai K., Yagi A. (2003). Comparison between the lambda response of eye-fixation-related potentials and the P100 component of pattern-reversal visual evoked potentials. Cogn. Affect. Behav. Neurosci..

[23] Eimer M., Velzen J.V., Driver J. (2002). Cross-modal interactions between audition, touch, and vision in endogenous spatial attention: ERP evidence on preparatory states and sensory modulations. J. Cogn. Neurosci..

[24] Brouwer A.M., Reuderink B., Vincent J., van Gerven M.A., van Erp J.B. (2013). Distinguishing between target and nontarget fixations in a visual search task using fixation-related potentials. J. Vis..

[25] Brouwer A.M., Hogervorst M.A., Oudejans B., Ries A.J., Touryan J. (2017). EEG and eye tracking signatures of target encoding during structured visual search. Front. Hum. Neurosci..

[26] Finke A., Essig K., Marchioro G., Ritter H. (2016). Toward FRP-based brain-machine interfaces—single-trial classification of fixation-related potentials. PLoS ONE.

[27] Shulman G.L., Pope D.L., Astafiev S.V., McAvoy M.P., Snyder A.Z., Corbetta M. (2010). Right hemisphere dominance during spatial selective attention and target detection occurs outside the dorsal frontoparietal network. J. Neurosci..

[28] Yamaguchi S., Yamagata S., Kobayashi S. (2000). Cerebral asymmetry of the “top-down” allocation of attention to global and local features. J. Neurosci..

[29] Mantini D., Corbetta M., Perrucci M.G., Romani G.L., Del Gratta C. (2009). Large-scale brain networks account for sustained and transient activity during target detection. Neuroimage.

[30] Hoffman E.A., Haxby J.V. (2000). Distinct representations of eye gaze and identity in the distributed human neural system for face perception. Nat. Neurosci..

[31] Haxby J.V., Hoffman E.A., Gobbini M.I. (2002). Human neural systems for face recognition and social communication. Biol. Psychiatry.

[32] Leveroni C.L., Seidenberg M., Mayer A.R., Mead L.A., Binder J.R., Rao S.M. (2000). Neural systems underlying the recognition of familiar and newly learned faces. J. Neurosci..

[33] Gobbini M.I., Haxby J.V. (2007). Neural systems for recognition of familiar faces. Neuropsychologia.

[34] Vilberg K.L., Rugg M.D. (2008). Memory retrieval and cortex: A review of evidence from a dual-process perspective. Neuropsychologia.

[35] Baccino T., Manunta Y. (2005). Eye-Fixation-Related Potentials: Insight into Parafoveal Processing. J. Psychophysiol..

[36] Schupp H.T., Junghöfer M., Weike A.I., Hamm A.O. (2003). Attention and emotion: An ERP analysis of facilitated emotional stimulus processing. Neuroreport.

[37] Chandra S.R., Patwardhan K., Pai A.R. (2017). Problems of Face Recognition in Patients with Behavioral Variant Frontotemporal Dementia. Indian J. Psychol. Med..

[38] Donato L., Scimone C., Alibrandi S., Abdalla E.M., Nabil K.M., D’Angelo R., Sidoti A. (2021). New Omics—Derived Perspectives on Retinal Dystrophies: Could Ion Channels-Encoding or Related Genes Act as Modifier of Pathological Phenotype?. Int. J. Mol. Sci..

[39] Donato L., Scimone C., Alibrandi S., Pitruzzella A., Scalia F., D’Angelo R., Sidoti A. (2020). Possible A2E Mutagenic Effects on RPE Mitochondrial DNA from Innovative RNA-Seq Bioinformatics Pipeline. Antioxidants (Basel).

[40] Scimone C., Alibrandi S., Scalinci S.Z., Trovato Battagliola E., D’Angelo R., Sidoti A., Donato L. (2020). Expression of Pro-Angiogenic Markers Is Enhanced by Blue Light in Human RPE Cells. Antioxidants (Basel).

